# Laryngoscope-Guided plasma radiofrequency ablation combined with incision/drainage for neonatal congenital pyriform Sinus Fistula with abscess: safety and minimally invasive approach

**DOI:** 10.3389/fped.2026.1862198

**Published:** 2026-06-29

**Authors:** Dongjihui Zhao, Yun Li, Binya Hu, Min Huang, Sijun Zhao, Guangliang Liu

**Affiliations:** Department of Otolaryngology-Head and Neck Surgery, Children’s Hospital Affiliated to Xiangya School of Medicine, Central South University (Hunan Children’s Hospital), Changsha, China

**Keywords:** abscess drainage, congenital pyriform sinus fistula, laryngoscope-guided, newborn, plasma radiofrequency ablation, recurrence prevention

## Abstract

**Objective:**

To evaluate the clinical safety, efficacy, and recurrence management of a combined surgical approach—neck abscess incision and drainage coupled with low-temperature plasma radiofrequency ablation under laryngoscope-guided suspension laryngoscopy, for neonatal congenital pyriform sinus fistula (CPSF) at the abscess stage.

**Methods:**

At Hunan Children’s Hospital, a retrospective analysis of 21 neonatal abscess-stage CPSF patients was conducted between January 2020 and January 2025. Operative time, blood loss, 24-hour NIPS pain score, vocal cord mobility, NFSS-8 swallowing score, hospital stay, and recurrence rate were the main metrics compared between the simple drainage group (*n* = 7) and the combined surgery group (*n* = 14) based on family request.

**Results:**

There were no group differences in baseline variables (gender, gestational age, birth weight, etc.) (all *P* > 0.05). In terms of surgery, the combined group experienced equal blood loss (*P* = 0.068), pain levels (*P* = 0.624), and a longer operating time (*P* < 0.001). A lower NFSS-8 score (11.87 ± 2.94 vs. 15.29 ± 4.01, *P* = 0.019) and more normal vocal cord movement (92.86% vs. 57.14%, *P* = 0.043) demonstrated greater short-term recovery. Hospital stays were comparable (*P* = 0.781), but the 12-month recurrence rate was lower (7.14% vs. 100%, *P* = 0.002).

**Conclusion:**

Combining neck abscess incision and drainage with Plasma Radiofrequency Ablation under Laryngoscope-guided is a safe and efficient treatment for congenital pyriform sinus fistula (CPSF) in neonates with abscess, with acceptable short-term functional recovery and a lower recurrence rate. This minimally invasive approach warrants further investigation in larger prospective, randomized studies.

## Introduction

Internal fistula holes are found within the pyriform sinus region of CPSF, which arises from residual epithelium caused by inadequate closure of the third and fourth pharyngeal pouches during development. CPSF continues to have a high misdiagnosis rate because of its intricate embryonic development mechanisms, wide range of anatomical variants, and subtle clinical presentation. Affected families bear high financial and psychological costs as a result of the ongoing therapy procedure. Neonatal onset is uncommon, and about 80% of cases appear during childhood. Neonatal presentations, however, frequently include a primary neck abscess that results in torticollis, hoarseness, and a high temperature. Stridor, respiratory discomfort, dysphagia, unilateral vocal cord paralysis, or even potentially fatal consequences like mediastinal abscess or pneumothorax might occur in difficult situations (1–6).

A “two-stage” strategy is used in traditional treatment: during the acute infection phase, sufficient antibiotics are used to control the infection; when abscesses form, an incision and drainage are made; after infection control, open surgery is performed to remove the fistula completely (7–9). Significant surgical trauma, complicated procedures, high complication risks (such as pharyngeal fistula and vocal cord paralysis), cosmetic issues owing to postoperative scarring, and consistently high recurrence rates are some of this approach’s major disadvantages. As of right now, there isn't a single accepted method for figuring out the best time and surgical indications for CPSF.

## Materials and methods

### Clinical information

In this retrospective cohort study, clinical data from 21 babies hospitalized at Hunan Children’s Hospital between January 2020 and January 2025 and diagnosed with CPSF in the abscess stage were methodically analyzed. Gender, gestational age at birth, birth weight, age at surgery, Apgar scores at 1 and 5 min, fistula side (left or right), duration of fever, respiratory distress interventions (such as oxygen therapy or intubation), and maximum diameter of the cervical abscess were among the baseline data that were taken from electronic medical records. Depending on parental treatment preferences, patients were divided into two groups: the simple drainage group (*n* = 7) and the combined surgery group (*n* = 14). It should be noted that this was a non-randomized, retrospective cohort. Allocation to the simple drainage group vs. the combined surgery group was based on parental preference rather than standardized clinical criteria, which may introduce selection bias and confounding. Accordingly, causal inferences regarding comparative efficacy cannot be drawn, and the results should be interpreted as hypothesis-generating. There were no statistically significant differences between groups in the baseline data (*P* > 0.05). This retrospective study was reviewed and approved by the Institutional Review Board of Hunan Children’s Hospital [Approval No. (HLCHLL-2025-226)]. Given the retrospective nature of the study and the use of anonymized clinical data, the requirement for written informed consent for participation was waived. However, written informed consent for surgical procedures and postoperative follow-up was obtained from the legal guardians of all patients.

1. Neonates under 28 days of age; 2. Clinical presentation of recurrent neck infection with subhyoid skin fistula formation; 3. Direct laryngoscopy showing a clear internal fistula opening at the base of the pyriform sinus; 4. Persistent infection (sustained fever >38.5 °C or C-reactive protein >20 mg/L) following ≥14 days of empirical broad-spectrum antibiotic therapy. In this neonatal cohort, this criterion referred to the cumulative duration of antimicrobial exposure​ prior to definitive surgical intervention, often involving multiple regimens and adjustments rather than continuous monotherapy. The decision to proceed to surgery was based on persistent clinical and laboratory signs of infection despite antimicrobial therapy.5. Imaging studies (neck ultrasound/enhanced CT/MRI) showing abscess formation (abscess diameter ≥1.5 cm); 6. Written informed consent from the legal guardian. Exclusion Standards: 1. Coagulation abnormalities (INR > 1.5 or platelets < 50 × 10³/L); 2. Concurrent congenital heart disease or severe respiratory diseases (e.g., bronchopulmonary dysplasia); 3. A history of radiation therapy or neck surgery; 4. Family refusal of general anesthesia or postoperative follow-up.

### The process of diagnosis

Every patient had increasing neck edema and soreness when they first arrived. Vital sign monitoring, full blood count + CRP tests, and neck palpation were all part of the initial assessment. Fiberoptic laryngoscopy, neck color Doppler ultrasound, and neck CT or MRI were carried out where clinically possible. Under supportive laryngoscopy, all instances showed a fistula opening at the base or apex of the pyriform sinus, confirming the CPSF diagnosis.

### Method of surgery

Ablation and Drainage Group Combined: Positioning and Anesthesia: Endotracheal intubation under general anesthesia; supine position with shoulders raised 15 degrees. Endoscopic Phase of Ablation: To reveal the afflicted pyriform sinus, a 4.0 mm diameter direct laryngoscope (Storz, Germany) was placed transorally. After identifying the internal fistula hole, an assistant compressed the cervical abscess to verify fistula drainage while the laryngoscope was supported with a support frame. The perifistulous region was disinfected with diluted povidone-iodine. A low-temperature plasma radiofrequency probe (mc-403, power settings: cutting level 4/hemostasis level 3) was inserted. The probe was directed laterally toward the thyroid cartilage lamina to avoid the anatomical course of the recurrent laryngeal nerve. Ablation was performed 1 cm inside the fistula orifice and extended 2–3 mm peripherally, confined to the perifistular mucosa and superficial submucosa. Continuous saline irrigation was applied throughout the procedure to minimize thermal spread. Ablation endpoint: There is no active bleeding, and a grayish-white staining on the fistula mucosa. Phase of neck drainage: Perform standard cleaning and draping after removing the laryngoscope. Aspirate a sample of pus using a 10 mL syringe. Along skin wrinkles, make a 1.5-cm transverse incision. Bluntly dissect the abscess cavity. Remove pus and necrotic tissue completely. Use saline, povidone-iodine, and 3% hydrogen peroxide in that order. Put a rubber drainage strip at the abscess cavity’s base. To finish the process, apply a sterile dressing using pressure bandaging.

Simple drainage group: Families in this group explicitly refused general anesthesia​ due to concerns regarding surgical risks, opting for local anesthesia drainage only. On the side of the neck that is afflicted, perform routine surgical skin preparation and draping. Aspirate and collect pus samples using a 10 mL syringe, then send them right away for microbiological culture and sensitivity testing. At the location of the most noticeable variation, a transverse incision measuring about 1.5 cm was made along the skin creases. Major arteries and nerves were carefully preserved during the blunt dissection of the abscess space. To enable pus to drain, the abscess septa were completely opened. Use saline, povidone-iodine, and 3% hydrogen peroxide in that order. Put a rubber drainage strip at the abscess cavity’s base. To finish the process, apply a sterile dressing using pressure bandaging.

Postoperative Management: Antibiotic Therapy: Depending on the results of the culture, either stick to the original antibiotic regimen or switch to more sensitive drugs. Drainage Tube Maintenance: Record drainage volume and irrigate drainage tubes every day with 20 milliliters of saline solution. Nutritional Support: After surgery, keep the nasogastric tube in place for at least 14 days before progressively switching to oral feeding. Pain management involves monitoring with the Neonatal Pain Assessment Scale (NIPS) and giving acetaminophen (10 mg/kg per dose) as necessary.

### Indicators of observation

Safety indicators include incision infection, pharyngeal fistula, voice cord paralysis, and intraoperative blood loss.2. Efficacy metrics: length of hospital stay, recurrence rate, and length of surgery;3. Functional evaluation: swallowing function (using the NFSS-8 scale); vocal cord mobility using fiberoptic laryngoscopy two weeks after surgery.V. Protocol for Follow-Up: Create uniform follow-up documentation. Timepoints for follow-up: 2 weeks, 1, 3, 6, and 12 months after surgery; Items of examination: Clinical evaluation includes swallowing function observation, neck palpation, fiberoptic laryngoscopy, and imaging tests (such as contrast-enhanced CT or MRI to assess closure of the internal fistula opening and neck ultrasounds every three months).

Recurrence is defined as any of the following circumstances: redness, swelling, and purulent discharge in the neck; imaging verification of the development of an abscess (diameter ≥1.0 cm); A laryngoscopy shows that the initial fistula orifice has reopened.

### Analysis of statistics

SPSS 21.0 was used for statistical analysis. Normality of continuous data was assessed using the Shapiro–Wilk test. Normally distributed data were expressed as mean ± standard deviation (x ± s). Intergroup comparisons of continuous variables were conducted using independent samples *t*-tests. Categorical variables were presented as frequencies and percentages [n (%)] and compared using Fisher’s exact probability test, given the small sample size and expected cell counts <5 in some categories. Statistical significance was defined as a two-tailed *P* < 0.05. Given the non-randomized nature of this study and the small sample size, the results are interpreted as hypothesis-generating, and causal inferences regarding treatment efficacy cannot be established.

## Results

### Comparison of baseline data

Gender distribution, gestational age at birth, birth weight, age at surgery, Apgar scores, fistula side distribution, proportion of patients with fever, proportion requiring intervention for respiratory distress, baseline inflammatory markers (white blood cell count and C-reactive protein levels), and abscess size did not differ statistically significantly between the groups (*P* > 0.05). Table 1 presents detailed facts.

**Table 1:** Comparison of baseline data between groups.

Variable	Combined Surgery Group (*n* = 14)	Simple Drainage Group (*n* = 7)	Statistic	*P*-value
Gender, *n* (%)			Fisher	0.482
Male	6 (42.86)	4 (57.14)		
Female	8 (57.14)	3 (42.86)		
Gestational Age (weeks,×± s)	39.35 ± 1.08	39.72 ± 0.95	t = 0.80	0.531
Birth Weight (kg,×± s)	3.18 ± 0.52	3.47 ± 0.42	t = 1.38	0.387
Apgar 1 min (points,×± s)	9.05 ± 0.85	8.72 ± 1.25	t = 0.63	0.684
Apgar 5 min (points,×± s)	5.72 ± 1.63	5.92 ± 1.95	t = 0.23	0.473
Fistula Side, *n* (%)			Fisher	0.628
Left	11 (78.57)	6 (85.71)		
Right	3 (21.43)	1 (14.29)		
Fever, *n* (%)	5 (35.71)	3 (21.43)	Fisher	0.598
Respiratory Distress, *n* (%)	6 (42.86)	4 (57.14)	Fisher	0.712
White Blood Cell Count (×10⁹/L,×± s)	15.82 ± 4.25	17.45 ± 5.10	t = 0.85	0.406
C-reactive Protein (mg/L,×± s)	42.60 ± 18.35	48.90 ± 22.15	t = 0.72	0.481
Max Abscess Diameter (cm,×± s)	4.42 ± 1.53	4.02 ± 2.18	t = 0.44	0.670

### No patients were lost to follow-up during the 12-month observation period

All recurrences were confirmed by a combination of clinical signs (neck redness, swelling, and purulent discharge) and imaging evidence (neck ultrasound or enhanced CT showing abscess formation ≥1.0 cm). Perioperative management, intraoperative infection rates, postoperative reactions, functional recovery, and long-term prognosis are just a few of the multidimensional indicators that were used in a thorough comparison of clinical outcomes between combined ablation and drainage procedures vs. drainage alone. Table 2 presents detailed facts.

**Table 2:** Comprehensive comparison of clinical outcomes between groups.

Variable	Combined Surgery Group (*n* = 14)	Simple Drainage Group (*n* = 7)	Statistic	*P*-value
Operative Time (min,×± s)	61.23 ± 13.89	35.14 ± 8.97	t = 5.19	<0.001
Intraoperative Blood Loss (mL,×± s)	5.15 ± 2.37	4.82 ± 3.05	t = 1.90	0.068
24-hour postoperative NIPS (points,×± s)	3.05 ± 1.12	2.86 ± 0.98	t = 0.40	0.624
Vocal Cord Movement, *n* (%)			Fisher	0.043
Normal	13 (92.86)	4 (57.14)		
Abnormal	1 (7.14)	3 (42.86)		
Postoperative 1-month NFSS-8 score (x ± s)	11.87 ± 2.94	15.29 ± 4.01	t = 2.48	0.019
Hospitalization Days (d,×± s)	20.32 ± 3.85	20.89 ± 4.19	t = 0.30	0.781
Recurrence at 12 months, *n* (%)			Fisher	0.002
Yes	1 (7.14)	7 (100.00)		
No	13 (92.86)	0 (0.00)		

The Neonatal Feeding and Swallowing Screen-8 (NFSS-8) is a validated tool for neonates; higher scores indicate poorer swallowing function. The Neonatal Infant Pain Scale (NIPS) was used for postoperative pain assessment. Bold values indicate *P* < 0.05.

During the 12-month follow-up period, no patients were lost to follow-up. A total of 7 patients were enrolled in the simple drainage group, and 14 in the combined surgery group. There was no statistically significant difference in intraoperative blood loss between the two groups (*P* = 0.068), while the combined surgery group had significantly longer operating times (*P* < 0.001). Postoperative assessment at 24 h showed comparable NIPS pain scores in both groups (*P* = 0.624).

In terms of short-term functional recovery, the combined surgery group achieved superior outcomes. The vocal cord normalization rate was 92.86% in the combined surgery group, compared with 57.14% in the simple drainage group (*P* = 0.043). In addition, the NFSS-8 swallowing function score was 11.87 ± 2.94 in the combined surgery group and 15.29 ± 4.01 in the simple drainage group (*P* = 0.019). There was no significant difference in the length of hospital stay between the two groups (*P* = 0.781).

Regarding recurrence, the 12-month postoperative recurrence rate was 7.14% (1/14) in the combined surgery group, markedly lower than 100% (7/7) in the simple drainage group (*P* = 0.002). Detailed recurrence timing revealed that the sole recurrence in the combined group occurred at 8.0 months postoperatively, presenting as mild neck redness without obvious abscess formation. In contrast, all seven recurrences in the simple drainage group occurred within 6 months postoperatively (range: 2.8–6.0 months; mean: 4.2 ± 1.1 months). These recurrences were characterized by significant neck abscess formation (≥1.0 cm on imaging) or purulent discharge and were confirmed by a combination of clinical signs and imaging evidence (neck ultrasound or enhanced CT).

## Discussion

2% to 10% of all branchial cleft malformations are caused by the uncommon condition known as CPSF. Less than 1% of newborns experience it. CPSF should be taken into consideration as a main differential diagnosis if prenatal ultrasonography or MRI shows a cervical cystic mass accompanied by increasing expansion during swallowing. Four of the twenty-one instances in this study had a cervical cystic tumor found during pregnancy. Open pharyngeal sinus fistula resection during the newborn period without an infection, however, raised worries from the families. Only four of the cases in this cohort were on the right side of the neck, despite the fact that over 90% of CPSF cases occur on the left (10).

Misdiagnosis rates are consistently high because of the distinct anatomy of CPSF and its considerable clinical variability. Determining the best time for surgery and creating appropriate treatment plans depends on an early and correct diagnosis. Although these tests are not completely reliable, imaging examinations such as color Doppler ultrasonography, neck CT, or MRI are advised for babies (8, 11, 12). By seeing the internal fistula opening, fiberoptic laryngoscopy helps verify the diagnosis. This method is an essential screening tool for suspected CPSF in neonates because of its simplicity, non-invasiveness, and lack of sedative requirements. However, in clinical practice, the internal opening within the pyriform sinus is frequently obscured during routine examination by variables including mucosal folds, tissue edema during acute inflammation, or scar closure surrounding the fistula opening. Fiberoptic laryngoscopy was used to definitively diagnose only three instances in this sample. Thus, the gold standard for verifying CPSF is still general anesthesia combined with supraglottic laryngoscopy. Using this technique, all 21 patients in this study had distinct fistula openings at the base of the pyriform sinus tip, yielding 100% diagnostic accuracy.

When there is no infection, CPSF usually manifests as a branchial cleft cyst during the newborn stage. Swallowing results in severe cystic distension because newborn fistulas have a broad aperture. Large cysts that are close to the airway have the potential to compress the trachea and cause hazardous symptoms like respiratory distress and stridor. CPSF should be highly suspected if a cystic mass on the left side of the newborn’s neck is seen to enlarge after swallowing (13–15). The infant’s primary symptoms during an acute infection are a painful lump on the left neck and recurring fever. Acute suppurative thyroiditis can cause diffuse neck swelling or possibly the formation of an abscess in certain circumstances. Recurrent or persistent fistulas often form after incision and drainage. Severe cases may develop into potentially fatal sequelae that require immediate medical attention, such as neck abscesses, high fever, torticollis, bacteremia, airway blockage (dyspnea, wheezing), dysphagia, vocal cord paralysis, mediastinal abscesses, and pneumothorax.

Notably, some kids continue to experience inflammatory reactions even after receiving regular antibiotic treatment. Clinicians are advised to proactively pursue surgical intervention at an appropriate stage based on disease progression in order to prevent worsening conditions and severe complications while minimizing the detrimental effects of prolonged antibiotic use, recurrent infections, surgical drainage, and frequent dressing changes on the child’s growth, psychological well-being, family quality of life, and medical burden. This proactive approach to treatment promotes overall family health, greatly improves patient outcomes, and enables curative resolution.

The only treatment for CPSF is still surgery, which focuses on removing all fistula tissue. In order to manage inflammation during an acute infection, traditional treatment procedures recommend giving enough antibiotics. If an abscess develops, an incision and drainage should be made, and open surgery should be carried out once the inflammation has subsided. While percutaneous catheter drainage may offer cosmetic advantages and reduce dressing-related discomfort, the presence of loculated, viscous pus in our cases favored open incision and drainage to ensure adequate evacuation. This staged approach has several disadvantages, including significant surgical trauma, complicated manipulation, high complication rates (reported at 5%–6% in the literature), noticeable postoperative scarring, and elevated recurrence rates, even though it allows for clear anatomical visualization and complete fistula excision (16). Endoscopic fistula closure has become a new therapeutic option in recent years due to advancements in minimally invasive procedures (17). This method uses laser, electrocoagulation, or chemical cauterization to accurately treat the internal fistula opening and surrounding mucosa, resulting in anatomical closure through scar repair. It has benefits, including little trauma, quick healing, no visible scars, and high success rates. But there are still issues, such as the possibility of heat harm, the difficulty of accurately regulating ablation conditions, and the surgeon’s high technical demands. Additionally, a crucial requirement for the success of surgery is comprehensive preoperative infection control (18). There remains controversy over performing internal fistula ablation during acute exacerbation or abscess stages, mainly due to severe local tissue edema and marked inflammatory reactions. Notably, CPSF in neonates progresses rapidly and may become life-threatening in a short time. Therefore, exploring minimally invasive surgical techniques applicable simultaneously during the abscess stage is of great clinical significance. For Plasma Radiofrequency Ablation, studies by Li Wanpeng (19) and Dou Qianwen et al. (20) showed recurrence rates of 0% and 5.8%, respectively. This method minimizes heat damage and reduces procedure time by operating at comparatively low temperatures (40–70 °C). Shorter hospital stays and no neck scars are possible with the plasma knife’s ability to penetrate 1 cm into the fistula for full-thickness ablation while maintaining hemostasis. Simultaneous abscess drainage is possible for patients in the abscess stage. In this study, combined cervical abscess drainage and low-temperature plasma ablation under suspension laryngoscopy were adopted for neonatal CPSF. All operations were performed under Laryngoscope-guided, targeting the fistula orifice at the base of the pyriform sinus. To reduce thermal injury, especially to the adjacent recurrent laryngeal nerve, the plasma probe was directed toward the lateral thyroid cartilage lamina, with ablation confined to the mucosa and superficial submucosa around the fistula orifice. Continuous normal saline irrigation was applied throughout the procedure to mitigate heat diffusion, and the ablation area extended 2–3 mm outward from the fistula orifice. The 12-month follow-up revealed a recurrence rate of merely 7.14% in the combined treatment group, compared with 100% in the group receiving abscess drainage alone. The difference was statistically significant (*P* < 0.05). The 100% recurrence rate observed in the simple drainage group may appear unusually high but is biologically plausible given the unique pathophysiology of neonatal CPSF. Unlike adults, neonates possess a relatively wide internal fistula opening and thin-walled tracts. Simple incision and drainage merely evacuates purulent collections without addressing the epithelial lining of the fistula. Consequently, persistent salivary leakage and bacterial colonization inevitably lead to recurrent infection and abscess formation. Our findings align with the traditional understanding that definitive treatment requires complete fistula obliteration. In conclusion, for neonates with CPSF presenting with neck abscess, the combination of laryngoscope-guided low-temperature plasma radiofrequency ablation and drainage was associated with a lower 12-month recurrence rate and feasible short-term functional recovery compared to drainage alone.

A major concern in non-randomized studies is selection bias arising from differences in baseline disease severity. In this cohort, although treatment allocation was based on parental preference, we found no significant differences in objective inflammatory markers between the groups. Specifically, the white blood cell count (15.82 ± 4.25 vs. 17.45 ± 5.10, *P* = 0.406) and C-reactive protein levels (42.60 ± 18.35 vs. 48.90 ± 22.15, *P* = 0.481) were similar between the combined surgery and simple drainage groups (Table 1). The severity of acute infection at presentation was comparable between the two groups, suggesting that the observed outcomes were unlikely to be substantially confounded by initial disease severity. Therefore, the markedly lower recurrence rate in the combined surgery group was mainly attributed to complete obliteration of the fistula tract rather than differences in baseline infection severity.

This study suggests routinely placing nasogastric tubes throughout the preoperative period based on clinical findings. For neonatal patients, we recommend extending nasogastric tube installation to two weeks or beyond, despite prior research generally recommending postoperative nasogastric feeding for one to two weeks. The following pathophysiological factors serve as the main foundation for this recommendation: Infants and newborns frequently experience gastroesophageal reflux. Infants and neonates frequently have gastroesophageal reflux. Complications include hoarseness, persistent laryngitis, and vocal cord irritation can result from the reflux of acidic stomach contents into the throat (21, 22).

Continuous nasogastric tube insertion before and after surgery successfully lessens gastric acid’s chemical irritation of the pharyngeal mucosa, promoting wound healing and dramatically reducing the chance of recurrence. The effectiveness of this strategy was confirmed by the fact that all 14 newborns in this trial who underwent combined ablation and drainage procedures kept nasogastric tubes for two weeks after surgery, with only one recurrence noted.

Both simple incision and drainage and combination ablation-drainage techniques cause postoperative discomfort when treating CPSF in neonates during the abscess phase, with mechanisms showing both similarities and differences. During abscess drainage, mechanical tension on surrounding tissues immediately stimulates nerve endings and initiates the transmission of pain signals, which is the common mechanism for pain in both operations. Concurrently, the enormous release of inflammatory mediators like prostaglandins and bradykinin is encouraged by the local inflammatory response brought on by surgical stress. These mediators increase sensitivity to pain, reduce nerve endings’ pain threshold, and worsen pain perception. Nevertheless, combined ablation drainage did not significantly increase discomfort (*P* = 0.624), even though it added a cryo-plasma fistula ablation step to the basic incision and drainage approach. This is explained by the advantages of its special mechanism. Low-temperature plasma ablation demonstrates accurate, less invasive thermal management from a thermal standpoint (23). With a penetration depth of no more than 1 mm, its thermal effects are strictly contained within the fistula wall. This successfully stops further pain stimuli brought on by significant tissue damage by reducing thermal diffusion to nearby healthy tissue. In terms of controlling inflammation, ablation effectively blocks the channel for persistent pathogen invasion by sealing the fistula right away. This lessens ongoing local infection stimulation, which lowers the generation of inflammatory mediators and relieves pain at the site. Moreover, 40–70 °C is the operating temperature for low-temperature plasma ablation. This minimally maintains normal nerve ending function in comparison to high-temperature operations, minimizing broad nerve degeneration that causes pain sensitivity and further lowering postoperative pain levels (24). A systematic review by Olsson et al. summarized the standardized application and reporting criteria of various neonatal pain scales (25).

One incidence of vocal cord impairment was recorded by the combined group, which may have resulted from the ablation site’s close proximity to the recurrent laryngeal nerve (fistulas in the pyriform sinus frequently adjoin the nerve’s route). Even with low temperatures, there is still a chance of slight stimulation or compression due to edema. Simple drainage, on the other hand, just treats the abscess and does not include the fistula area, preventing further damage to the larynx. Reasons for the Combined Group’s Better Swallowing Performance. The fundamental mechanism that fistula ablation lessens the effect of recurrence/residual lesions on swallowing accounts for the combined group’s superior swallowing function: Only the abscess is removed by simple drainage; the fistula is left untreated. Following surgery, the pharyngeal mucosa may be continuously irritated by fistula secretions and local adhesions, which could impede the recovery of swallowing. By sealing the fistula at its origin, the combined method removes any remaining lesions, which reduces pharyngeal inflammation and speeds up the repair of swallowing-related muscle and mucosal function.

CPSF abscesses in newborns can be fatal and frequently develop quickly, making laryngoscopy the gold standard for diagnosis. Although the combined procedure requires a longer operative time than isolated drainage, it was associated with improved short-term rates of normal vocal cord movement and swallowing function recovery, while significantly lowering the risk of postoperative recurrence. The minimally invasive nature, high success rate, and low recurrence rate of this method represent its key strengths. Simultaneously, systematic anti-reflux therapy and preoperative nasogastric tube implantation are essential steps to lower recurrence and justify clinical advancement.

This study has several limitations that warrant careful consideration. First, its single-center, retrospective, and non-randomized design inherently introduces selection bias. Treatment allocation was based on parental preference​ rather than standardized clinical criteria, and factors such as infection severity, airway compromise, and the need for ICU support likely acted as confounding variables. Second, the small sample size (14 vs. 7 patients) limits statistical power and reduces the generalizability of our findings. Third, blinding was not feasible due to the visible absence of neck scars in the combined surgery group, which may have introduced detection bias. Despite these limitations, this study provides preliminary evidence supporting the feasibility of a minimally invasive approach for neonatal CPSF. Future prospective, multicenter studies with larger cohorts and randomized designs are required to validate these findings and to establish definitive surgical indications.

In conclusion, laryngoscope-guided low-temperature plasma radiofrequency ablation combined with neck drainage represents a feasible and effective minimally invasive option for neonatal CPSF during the abscess stage. Compared with drainage alone, this combined approach was associated with improved short-term functional recovery and a significantly lower risk of recurrence. While systematic anti-reflux management and prolonged nasogastric tube placement are recommended to optimize outcomes, larger prospective studies are warranted to confirm these findings.

## Data Availability

The raw data supporting the conclusions of this article will be made available by the authors, without undue reservation.
